# Melanoma M (Zero): Diagnosis and Therapy

**DOI:** 10.1155/2013/616170

**Published:** 2013-04-11

**Authors:** Marco Rastrelli, Mauro Alaibac, Roberto Stramare, Vanna Chiarion Sileni, Maria Cristina Montesco, Antonella Vecchiato, Luca Giovanni Campana, Carlo Riccardo Rossi

**Affiliations:** ^1^Melanoma and Sarcoma Unit, Veneto Institute of Oncology, IOV IRCCS, 35128 Padua, Italy; ^2^Dermatology Unit, University of Padua, 35128 Padua, Italy; ^3^Department of Medical Diagnostic Sciences and Special Therapies, University of Padua, 35128 Padua, Italy; ^4^Medical Oncology Unit, Veneto Institute of Oncology, IOV IRCCS, 35128 Padua, Italy; ^5^Institute of Anatomopathology, University of Padua, 35128 Padua, Italy; ^6^Department of Surgical Oncological and Gastroenterological Sciences, Padua University, 35128 Padua, Italy

## Abstract

This paper reviews the epidemiology, diagnosis, and treatment of M zero cutaneous melanoma including the most recent developments. This review also examined the main risk factors for melanoma. Tumor thickness measured according to Breslow, mitotic rate, ulceration, and growth phase has the greatest predictive value for survival and metastasis. Wide excision of the primary tumor is the only potentially curative treatment for primary melanoma. The sentinel node biopsy must be performed on all patients who have a primary melanoma with a Breslow thickness > 1 mm, or if the melanoma is from 0,75 mm to 1 mm thick but it is ulcerated and/or the mitotic index is ≥1. Total lymph node dissection consists in removing the residual lymph nodes in patients with positive sentinel node biopsy, or found positive on needle aspiration biopsy, without radiological evidence of spread. Isolated limb perfusion and isolated limb infusion are employed in patients within transit metastases with a rate of complete remission in around 50% and 38% of cases. Electrochemotherapy is mainly indicated for palliation in cases of metastatic disease, though it may sometimes be useful to complete isolated limb perfusion. The only agent found to affect survival as an adjuvant treatment is interferon alpha-2. Adjuvant radiotherapy improves local control of melanoma in patients at a high risk of recurrence after lymph node dissection.

## 1. Introduction

Since the 1960s, malignant melanoma incidence has increased in Caucasian populations, and consequently this neoplasm has become one of the most frequent cancer in fair-skinned populations. Melanoma is now regarded as the fifth most common cancer in men and the sixth most common cancer in woman in the United States. The highest recorded incidence of melanoma worldwide is in Queensland (Australia) with an incidence equal to 55.8/10^5^/annum for males and 41.1/10^5^/annum for females. Reported incidence rates vary for Europe and are the highest in Switzerland and Scandinavian countries. In Europe, there is a north-south gradient in incidence rates with the highest rates in northern countries and the lowest ones in the southern countries. This is probably due to both the increased protection against UV exposure of the highly pigmented skin of southern Europeans and the different pattern of sun exposure (chronic in southern Europeans, intermittent in northern Europeans). In parallel with the increased incidence rate, there is an increase of melanoma related-mortality. The median age at the time of diagnosis is 57 years, and the incidence increases after the age of 25 until 50 years. Males are approximately 1.5 times more likely to develop melanoma than females. Favored sites of occurrence of cutaneous melanoma are sex dependent: the back for men and the arms and legs for women [1].

In Japan, the male-to-female ratio ranged from 1 : 0.97 to 1 : 1.14, and the survival rate of female patients is higher than that of male patients (the 140-month survival rate was 70.6% in females and 60% in males). Age distribution reached a peak at around 60 years. The sole of the foot was the most common site of melanoma in both males and females, whereas the rare melanomas on the lower limbs are generally observed in females [2].

Cutaneous melanoma (CM) is the most common cause of mortality among skin cancer in Caucasian populations (incidence rates per 100,000 patient years vary between 21.9 in the United States to 55.9 in Australian males). In contrast, the incidence of melanoma in Asia is significantly lower: incidence rates of 0.2 to 0.5 per 100,000 patient years. In addition, the most common histological subtype in Asians is acral lentiginous melanoma 3 (ALM) which accounts for approximately 50% of all cases, compared to Caucasians populations where it constitutes only 2% to 3% of all cases. The Asian melanoma patients have thicker tumors with advanced disease state at presentation, resulting in greater morbidity and mortality. The five and ten years survival rates for Asian patients with primary ALM without metastasis are 80,3% and 67,5% compared to 91,3% and 87,5% in the United States [3]. The melanoma incidence in black skin is 3,4 for 100.000 [4].

The incidence rate of this disease varies widely in relation to the race. White populations have an approximately 10-fold greater risk of developing cutaneous melanoma than black, Asian, or Hispanic populations. However, both white and African American populations have a similar risk of developing plantar melanoma, and noncutaneous melanomas (e.g., mucosal) are more common in nonwhite populations.

Artificial UV exposure, psoralen-UVA, size of nevi, some cancer syndromes, and certain phenotypic characteristic may play a role in the development of CM [5].

The development of CM results from complex interaction between mutations in various genes and constitutional and/or inherited factors combined with environmental factors, mainly UV-radiations. A certain degree of consensus has been established on risk factors, diagnostic procedures (i.e., biopsy), treatment of the primary melanoma, and prognostic factors, although several diagnostic and therapeutic aspects are still a matter of discussion. The variability observed among different clinical guidelines proposed often reflects the opinions of experts which are based on their own experience more than on the literature evidence. This paper based on Medline, Embase, and Cochrane databases could represent a valid tool for the daily clinical practice, as it critically outlines the most recent evidence emerging from the literature in the light of the experience of a multidisciplinary group in charge of diagnosis and treatment of patients with melanoma.

Distant metastatic melanoma (stage IV) confers a 5-year survival of only 5%–10% and a median survival of 6 to 10 months, depending on the site of metastasis. Advanced melanoma spreads in an unpredictable fashion with widespread metastasis to any organ but in particular to skin, lung, brain, liver, and small bowel. The most effective therapeutic approach for stage IV melanoma is surgical resection. The rationale for surgical resection of stage IV melanoma is multifactorial: the surgical resection limits disease and interrupt the metastatic cascade, restore immune function with inhibition of metastatic progression, and does not preclude systemic therapy. The advent of new chemotherapies makes the role of surgical resection more relevant [6]. This part of melanoma treatment is very complex and a better understanding of tumor biology is the key to determining the correct sequence of therapy. For this reason, it was excluded from this review.

## 2. Aetiology

The most important environmental risk factor for CM is intermittent exposure to UV radiations, although nodular melanoma (NM) and ALM appear to have little relationship to sun exposure [7]. The main host risk factors are the number of melanocytic nevi on the skin, skin type, family history, and genetic susceptibility. Approximately 25% of melanoma cases occur in conjunction with a preexistent nevus; moreover, the total nevus count is positively correlated with melanoma risk. Patients with more than 100 nevi have a 7-fold increase in risk for melanoma. Giant nevi (>20 cm) are associated with a higher risk of melanoma. CM which develops on the site of preexistent nevi is usually located on the trunk [8].

Mutations or epigenetic silencing of cyclin-dependent kinase inhibitor 2A (CDKN2A or p16) are common genetic abnormality in patients with family history of melanoma. Moreover, over two-thirds of melanomas have activating mutations in B-RAF gene, leading to constitutive activation of the B-Raf/MKK/ERK signaling pathway. Melanomas on skin without chronic sun-induced damage had frequent mutations in BRAF, on the contrary acral and mucosal melanoma and melanoma on the skin with chronic sun damage frequently are characterized by Kit mutations. These mutations are responsible of cancer cell behavior through mechanisms that are still to be defined [10]. Furthermore, PTEN (phosphatase and tensin homolog) deleted on chromosome 10 can be found mutated, deleted, or epigenetically silenced in melanoma [11]. Patients with family cancer syndromes (familial retinoblastoma, Li-Fraumeni cancer syndrome, and Lynch syndrome type II) show higher risk of developing melanoma. One percent to eight percent of patients with prior history of melanoma will develop multiple primary melanomas [12]. Red hair, fair skin, numerous freckles, light eyes, sun sensitivity, and an inability to tan raise the risk of developing melanoma by approximately 50%. In particular, Melanocortin 1 receptor (MCR1) variants seems to be associated with red hair color phenotype and melanoma [13].

## 3. Diagnosis

### 3.1. Skin Self Examination

Skin self-examination has great potential as a simple, convenient method of screening for CM and precancerous lesions. Before the 1980s, melanomas were often recognized by identifying clinically macroscopic features; often they were detected in advanced stages when they appeared large, ulcerated, and vegetating. The need to educate physicians and patients for early melanoma recognition leads to the development of the ABCDE criteria. ABCDE is an acronym for asymmetry, border irregularity, colour alterations, diameter >6 mm, and evolution. These criteria were intended to be a simple tool to alert both patients and nondermatologists healthcare professionals in differentiating common melanocytic lesions from suspicious pigmented lesions. Adopting this criteria, the sensitivity of self skin examination ranges from 57% to 90% and specificity from 59% to 90% [14]. Other clinical approaches have been developed to enhance early diagnosis, such as the Glasgow 7-point Checklist, which includes 3 major criteria (change in size, shape, and color) and 4 minor criteria (sensory change; diameter of 7 mm or greater; and the presence of inflammation, crusting, or bleeding) [15]. Another suspicious feature to be considered is the Ugly Duckling sign. It is based on the perception that a pigmented lesion looks different from all of its neighbours.

### 3.2. Dermoscopy

Noninvasive diagnostic technique for *in vivo* observation of the skin, this device uses optic magnification to allow the visualization of morphological structures that are not visible to the naked eye. There are some general dermoscopic criteria which may help in the diagnosis of malignant melanocytic lesions. These criteria are summarized in Table 1. Furthermore, several acral melanocytic lesions have been described on the basis of dermoscopic features described by Argenziano et al. [9]. These criteria may help in the diagnosis of acral melanoma which represents 50% of melanoma cases in Asian population [16].

### 3.3. Total Body Photographic Images and Short Term Surveillance

Some melanomas can neither be diagnosed by naked eye nor dermoscopically. It is possible to create images that can be electronically captured, archived, retrieved, and compared. In this way, it is possible to detect subtle changes during the first stages of melanoma development.

### 3.4. Reflected Confocal Microscopy (RCM)

RCM allows noninvasive examination of native skin in real-time at a nearly histological resolution. The reflectance confocal microscope emits a near-infrared, coherent laser beam by which the human skin is illuminated. Some of the potential advantages are improvement of diagnostic accuracy, improved assessment of dermoscopic histological correlation, in vivo biopsy side selection, surgical margin assessment, and response control of conservative therapies in skin disease [17].

### 3.5. Biopsy

Every suspected pigmented lesions should be photographed and then undergo excisional biopsy for histological examination. Under local anaesthesia, a visual clear margin of at least 2 mm should be included, with a few millimeters of subcutaneous fat [18]. Incisional biopsy should only be used in case of lesions too large to be removed completely or in particular anatomical regions (e.g., the ear, nose, and face). If the material obtained proves insufficient, the biopsy should be repeated [19]. Incisional biopsy does not affect patient prognosis [20].

Shave biopsy is not recommended because it interferes with the histological diagnosis and the assessment of Breslow thickness [21].

If a melanoma of the nails is suspected, it may be necessary (depending on the site of the lesion) to partially or totally remove the nail plate and take multiple biopsies of the nail bed and/or matrix [22].

### 3.6. Histology

Pathologic diagnosis of cutaneous melanoma is based on the assessment of many histologic features such as asymmetry, poorly defined borders, lack of maturation, cytological atypia, and presence of mitoses in the deep part of the dermal component. None of these criteria is itself absolute, but each criterion should be, case by case, carefully weighed and correlated with clinical data. Although the majority of melanocytic lesions can be easily diagnosed, there is a challenging subset of cases, for which, even with the most rigorous application of the diagnostic criteria, it is very difficult, if not impossible, to ascertain the diagnosis [23] (Table 2).

## 4. Approach for Staging

### 4.1. Imaging

A problem currently debated in the literature concerns the indications and the limits of instrumental investigations used for CM staging. Based on our experience and on the data in the literature, we would recommend our approach showed in Table 3.

The indication for ultrasound of the lymph node drainage basins in patients with cutaneous melanoma is still controversial, mainly because some consider this test excessively operator-dependent [24]. The prevailing opinion nowadays seems to be that ultrasound is highly effective and inexpensive in the early identification of lymph node metastases. In our experience, the sensitivity and specificity of ultrasound were, respectively, 92% and 90%, using the parameters suggested by Vassallo. More recently, other authors have reported a higher sensitivity and specificity, of 99.2% and 99.3%, respectively, after associating ultrasound with the echo-color Doppler assessment of lymph node vascularization [25]. When metastatic disease is suspected in a lymph node, ultrasound with fine-needle aspiration cytology (FNAC) achieves a sensitivity of 92% and a specificity of 100% [26] (Table 3).

Other diagnostic instrumental investigations are warranted only in the event of clinical suspicion or dubious findings: Computed Tomography (CT), Magnetic Resonance Imaging (MRI) [27], and Fluorine-18-2-Fluoro-2-Deoxy-D-Glucose Positron Emission Tomography and Computed Tomography (PET-CT). Suspected distant metastases must also be investigated using FNAC or surgical biopsy. Leung et al. [6]. In conclusion, the literature does not reveal clear guidance on staging. In general for melanoma in situ is sufficient skin examination, ultrasound in association with FNAC is sensitive and specific. The role of the Rx chest and liver ultrasound is controversial in melanoma T2-4a, while there is much more consensus in total body CT and PET in cases of melanoma T4b. The LDH is useful in patients with metastases.

### 4.2. Sentinel Lymph Node Biopsy (SLNB)

SLNB is a surgical minimally invasive procedure for identifying patients with metastases in clinically and radiologically negative lymph nodes.

The MSLT-1 trial demonstrated that sentinel lymph nodes (SLNs) are tested in 95% of patients with a percentage of false negatives amounting to 17.6% and a very low rate of local complications (5%). The interim analysis of the results of this study globally revealed no survival advantage for patients who underwent SLNB, although patients with a Breslow thickness between 1.2 and 3.5 mm seemed to have a longer disease-free survival than the group of patients in which the biopsy was not performed. The analysis also showed that patients found positive on SLNB and who underwent lymph node dissection (LND) had a better global survival rate than those undergoing lymph node dissection only after developing clinically-evident metastatic nodes. The MSLT-1 did not consider patients with melanoma with a Breslow thickness <1 mm or >4 mm [28]. Three recent retrospective studies found that the percentage of positive SLN among patients with melanomas with a Breslow thickness <1 mm was in the range of 2% to 5%, and the cases involved had high Clark levels, high mitotic index, and a young age [29]. The probability of positive SLN in cases of melanoma with a Breslow thickness >4 mm accounts to 30%–40% [12].

The SLNB should be considered on all patients who have a primary melanoma with a Breslow thickness >0,75 mm, or if the melanoma is <0,75 mm thick but it is ulcerated and/or the mitotic index ≥1. Additional risk parameters are young age, Clark levels IV-V, and extensive regression. In about 5% of patients it is impossible to identify the SLN.

In conclusion, the relevance of evaluation of SLN and the correlation with the prognosis must be defined, and it is unclear whether SLNB improves local control of lymph node basins. The patients should be aware that SLNB is a staging procedure without proven therapeutic value; it is possible to fail to find SLN or to have a false negative results. Finally, actually, SLNB is increasingly used with adjuvant therapy clinical trials [30].

## 5. Clinical Staging

The clinical staging is based on history and physical examination including locoregional area and draining lymph node, complete skin examination, histopathologic microstaging, imaging, and SLNB. At the end of clinical staging, the patients can be categorized into three groups: stage I-II localized disease, stage III regional disease, and stage IV metastatic disease.  Table 4 shows a synthetic description of the last version AJCC melanoma staging system.

## 6. Surgical Treatment

### 6.1. Wide Excision (WE) of the Primary Tumor

Surgery is the only potentially curative treatment for primary melanoma. The surgical standard requires the *enbloc* resection of the scar of the primary melanoma with the surrounding healthy skin and subcutaneous tissue down to the fascia. The muscle fascia is not removed, unless it has already been infiltrated, because its removal does not affect the local recurrence rate, while it has far worse cosmetic sequelae. The aim of this treatment is to achieve local disease control.

The extent of the margins currently depends on the maximum (Breslow) thickness of the primary melanoma. A melanoma with a higher Breslow thickness is treated with wider excision margins than a melanoma of lower thickness.

Based on the results of the controlled clinical studies conducted to date and on a recent meta-analysis, sufficiently WE margins should not be more than 2 cm [31].

The recommended WE margins usually are 5 mm for melanoma in situ, 10 mm for melanoma ≤1 mm to 2 mm, and 20 mm for melanoma ≥2 mm [32]. In the case of lesions involving the face, the excision margins cannot always comply with the recommendations, given the need to reconcile oncological radicality with a satisfactory reconstruction.

### 6.2. Lymph Node Dissection (LND)

The most common sites of metastases from cutaneous melanoma are the locoregional lymph nodes (laterocervical, axillary, inguinal). Total LND consists in removing the residual lymph nodes as well as the SLNB, or the node found positive on needle aspiration biopsy, in patients with no radiological evidence of spread. Some studies have found at least one other lymph node positive for metastatic disease from melanoma in 20% of lymph node dissections.

The lymphatic stations most often involved are the inguinal, axillary, and laterocervical; the epitrochlear and popliteal are affected much more rarely. The other superficial lymph node stations are unlikely to be affected by metastatic disease. The neck dissection includes nodal levels I–V, with superficial parotidectomy if melanoma is located between zygoma and mastoid or for positive node in parotid gland. Some authors modified the neck dissection preserving level I and parotid gland for nodal metastases at level V. Axillary lymphadenectomy involves excision of three levels of axillary nodes. The groin dissection includes superficial and deep inguinal nodes and ilio-obturator nodes [33].

As a quality control measure in relation to the adequacy of lymph node dissection, it is important to provide an accurate description of the limits of the dissection and of the lymphatic stations involved [30].

Although they have yet to be validated, other parameters of LND quality could include: the N-Ratio (the number of lymph nodes found positive out of the total lymph nodes removed) [34] and the minimum number of lymph nodes considered adequate (10 for axillary dissections, 7 for inguinal dissections, and 20 for laterocervical dissections) [35].

Adequate LND is associated with a survival rate of 50% or more at 10 years among cases with only one positive lymph node, while the survival rate drops to 30% if two or three lymph nodes are metastatic. Extranodal metastases are also associated with a low survival rate [36]. Some surgeons are started with laparoscopic dissection of groin, and ilio-obturator node, but actually the data are very poor for an initial analysis.

## 7. Locoregional Treatment of Metastases Intransit

Metastases intransit are biologically indistinguishable from local recurrences. The clinical distinction is conventionally based on the distance of the nodule from the primary melanoma. The metastases intransit are defined as any skin or subcutaneous metastases that are >0,3 mm with diameter >0,05 mm from the primary lesion but are not beyond regional basin. Clinically, they present as single or multiple nodules that may be located in the epidermis, dermis, or subcutaneous fat. The incidence of local recurrences and metastases in transit is around 3%.

Patients with a single metastatic nodule should undergo surgical resection. Those with numerous nodules should receive other locoregional therapies, for example, hyperthermic isolated limb perfusion (HILP), isolated limb infusion (ILI), or electrochemotherapy (ECT). These therapies enable local disease control but fail to influence the prognosis: the 5-year survival of these patients is approximately 30%. Selected patients suitable for HILP or ILI should be referred to a specialized melanoma centre. The adjuvant systemic chemotherapy is ineffective. Radiation therapy is inefficient in controlling regional disease.

### 7.1. Surgical Excision

This is indicated, ensuring histologically negative margins, for patients with a single nodule, especially if the melanoma has favorable prognostic features. It may also be advisable to perform SLNB in such cases because the percentage of occult secondaries is high.

### 7.2. Hyperthermic Isolated Limb Perfusion

HILP is indicated for unresectable metastases in transit or bulky melanoma, and it is recommended as a first therapeutic option in cases with no evidence of remote metastases. This technique was developed by Creech and Krementz in 1957 for the purpose of administering high doses of one or more drugs to a particular body region to treat locally advanced neoplastic disease, while maintaining a very low systemic toxicity. Melphalan is the reference drug, used at doses of 10–13 mg/L (L of limb volume). Administering this drug under moderately hyperthermic conditions (39–41°C) achieves global response rates at 2 months of 80%–90%, complete responses in around 50% of cases, and a 5-year survival rate of 28.5% [37]. The side-effects (toxicity) for the limb concerned are evaluated according to the Wieberdink scale. With accurate monitoring of the drug's leakage from the perfusion circuit into the systemic circulation (which must be kept under 10%), the systemic toxicity of this treatment is generally very modest.

In the case of bulky melanoma (more than 15 metastatic lesions or one or more tumor lesions with diameter >3 cm) or failure to respond to a first perfusion, TNF-*α* may be associated with Melphalan (without significant increase toxicity), achieving a complete response in 58% of such cases, as opposed to 19% obtained with Melphalan [38].

### 7.3. Isolated Limb Infusion

ILI is indicated for recurrence after HILP or for patients with poor general conditions. This technique has been developed and implemented in the early 1990s by Kroon and Thompson at the Sidney Melanoma Unit. This technique is simplified and minimally in invasive respect to HILP. ILI is a low flow HILP, performed in hypoxic conditions (without oxygenation of the perfusate) via percutaneously placed catheters. Melphalan at doses of 7,5 mg/L of tissue and Actinomycin-D at doses of 7,5 *μ*/L of tissue are the reference drugs. ILI achieves complete response rate in 38% and partial response rate in 46% of cases. The 5-year survival rate is the same of HILP. The toxicity for the limb can be erythema and oedema (developing within 24 hr), intense inflammation surrounding the in transit metastases (within 48 hr), and superficial desquamation of the skin (after 3 weeks). In general, the regional toxicity seen following ILI is higher than HILP, although very rare is grade IV of toxicity, and no grade V toxicity has been observed [39].

### 7.4. Electrochemotherapy

The main indication for ECT is for palliation in cases of metastatic disease, though it may sometimes be useful to complete ILP. ECT is a repeatable treatment based on the local application of short electrical pulses that locally modify the cells' electromagnetic field, enabling a larger quantity of the drugs (bleomycin or cisplatin) to enter the target area and consequently induce necrosis.

In various case series, there are reports of complete responses in 60%–70% of the target nodules after repeated treatments. The treatment is generally conducted under local anesthesia and/or sedation. The bleomycin can be injected into the tumor or administered as an intravenous bolus depending on the number of metastatic nodules [40].

In conclusion, the HILP remains the treatment of choice for metastases in transit because it has the most high rate of complete responses. The ILP is an alternative to HILP in patients fragile and/or with disease that is distal to the middle third of the thigh; the ECT has the advantage that it can be applied in any part of the body and is an alternative in the event of failure of other therapies, can complete the HILP treatment, and can improve also the quality of life.

## 8. Adjuvant Radiotherapy

Adjuvant radiotherapy is a radiation therapy after lymph node dissection for patients who are at a moderate to high risk for regional relapse.

The literature review demonstrates that adjuvant radiotherapy improves local control of melanoma in these patients after lymph node dissection, but it does not change the patient's survival curve [41].

The most common indications are as follows:

1. complementary treatment after surgery where there are doubts concerning the oncological radicality of the procedure; the dosage is 6–9 Gy per session, up to 30–50 Gy;
2. complementary treatment after surgery in patients at high risk of local recurrence;
3. complementary treatment after laterocervical neck dissection, although there are divergent opinions on its usefulness for local disease control, and no randomized studies have been conducted on this issue.

## 9. Adjuvant Immunotherapy

Adjuvant therapy is indicated for those postoperative patients at high risk of recurrence and/or metastases, as patients with melanoma stage III and patients with node-negative disease with a high risk of recurrence, that is, deep primary tumors (T3b, T4a/b).

### 9.1. Interferon (INF)

Recombinant interferon and pegylated interferon alpha are the only approved drugs with a statistically significant effect on relapse-free survival (RFS) proved in randomized studies and confirmed by a meta-analysis, which reports a 13% reduction on the 5-year recurrence rate and an 11% reduction on the risk of death; interestingly, when analyzed by a subgroup, no particular IFN-*α* regimen, IFN-*α* type, TNM disease stage, or study design conferred any statistically significant differences in overall hazard ratio estimates [42]. A similar effect has been reported in a systematic review which described a reduction in the risk of recurrence by 17% after high dosages of INF, 12% for intermediate and 13% for low dosages, with a greater effect in patients with ulceration and smaller tumor burden (stage IIB and III) [43]. Significant treatment-related adverse events (AEs) favored the investigation of low-dose IFN-*α* given for a longer duration in the aim to improve the toxicity profile. In the only study comparing HDI and LDI versus observation (ECOG 1690), LDI was associated with a reduced fraction of grade 3/4 AEs compared to HDI (1 (0.5%) versus 17 (8.0%) grade 4 AEs), although RFS was significantly improved in the HDI population versus observation (HR = 1.28, *P* = 0.025) while LDI failed to achieve statistically significant durable improvement in RFS. Issues still debated, regarding the use of adjuvant IFN, are (a) optimal treatment duration related to its tolerance, (b) dose-efficacy ratio in individual patients, (c) identification of predictive and prognostic markers, (d) relevance of the administration route, (e) significance of the development of autoimmunity and its relationship with the efficacy, and (f) prospective evaluation of ulceration.

### 9.2. Vaccines

Melanoma vaccines have been extensively investigated in the hope of eliciting durable clinical responses with minimal additional toxicity.

Gangliosides are sialic acid-containing glycosphingolipids that are overexpressed on the surface of melanocytic cells. GM2 with bacillus Calmette-Guerin (bCG) as an adjuvant or combining it with the keyhole limpet hemocyanin (KLH) hapten and a QS21 adjuvant effectively induces antibody responses to GM2. Both approaches were tested in the phase III setting in E1694 and EORTC 18961 against HDI and observation, respectively. Neither failed to demonstrate any RFS/OS benefit for vaccine therapy [44].

Seven randomized trials of adjuvant allogeneic melanoma cell-based vaccines have been conducted—none of which have suggested any survival benefit.

Canvaxin, a polyvalent vaccine studied in stage III melanoma patients, in a retrospective study, was suggested as a median and a five-year OS advantage in vaccinated patients in respect to nonvaccinated patients. However, when compared in a phase III RCT for resected stage III/IV melanoma against BCG vaccination, it failed to improve either DFS or OS with survival being worse (5% in stage IV and 9% in stage III) in the Canvaxin arm. Among vaccination approaches, the most appealing under evaluation is the MAGE-3A recombinant protein combined with the adjuvant system AS15. Mage 3A antigen is expressed in approximately 50%–60% of patients with melanoma in stage III, and in previous phase I and II studies the recombinant protein showed immunological and clinical effect in patients with unresectable stages III and IV A. The clinical effect seemed related to a specific tumor-host genomic profile, and the tolerance to the treatment was excellent compared with other drugs or with the standard adjuvant treatments commonly used for solid tumors (breast, colon, gastric, and lung cancer) [45].

The Derma trial comparing Mage 3A vaccination with placebo in melanoma patients with macroscopic stage III disease completed the accrual in June 2011, the results are awaited in the next year.

### 9.3. Immune-Targeted Drugs

The exciting long-term survival benefits seen in patients with metastatic melanoma treated with CTLA-blocking drug, ipilimumab, have raised expectations that this therapy may have even greater benefits in the adjuvant setting.

Ipilimumab seems particularly interesting for its ability to produce long lasting responses and an increase in long-term survival, this could translate in a definitive curative effect.

The study EORTC 18071, comparing ipilimumab with placebo in stage III melanoma patients, completed the accrual in June 2011. The results are expected within 2014.

### 9.4. Target Drugs

In patients harboring BRAF mutations, the unequivocal clinical benefit obtained by BRAF inhibitors (vemurafenib or dabrafenib) in respect to Deticene in the treatment of advanced disease raised the question of the opportunity of testing the efficacy in the adjuvant setting. However, the rapidly acquired resistance and the possibly long-term toxicity of RAF pathway inhibition in other tissues suggested caution in planning the adjuvant studies [46].

## 10. Followup

Monitoring of patients after treatment is usually undertaken for the purpose of ensuring an early diagnosis of melanoma relapse and/or second melanoma [47]. However, no studies have been conducted to compare different follow-up protocols and determine their impact on patient survival. Data from the literature indicate that less intensive follow-up is appropriate for patients with melanoma with Breslow <1 mm and more intensive surveillance for patients with greater Breslow thickness, particularly during the first three years as in these years the highest percentage of recurrence can be observed (95%) [48]. However, no clinically important results about the effects of more- or less-intensive surveillance for early diagnosis of recurrent melanoma have been reported [49]. We generally recommend instrumental follow-up only for patients with Breslow thickness >1 for the first 5 years. To this regard, please see the chapter on the imaging of melanoma.

## Figures and Tables

**Table 1:** Dermoscopic criteria for diagnosis of melanoma [9].

	Naevus	Melanoma
Pigment network	Reticular pattern with brown network small symmetrical holes and thin network lines	Black, brown, or gray network irregular holes and thick lines irregularly distributed and ending abruptly at the periphery
Dots/globules	Regular in size and shape and evenly distributed	Irregular dots and globules for the shape, size
Streaks	Regular and symmetrical and typical of Spitz or Reed nevi	Irregular and unevenly distributed
Irregular pigmentation	Not present	Black, brown, and gray pigmented areas with irregular shape and/or distribution
Regression structure	Not present	White scare-like areas, blue areas, or a combination of both
Blue-whitish veil	Not present	Confluent, irregular, and structureless area of whitish-blue diffuse pigmentation associated with pigmented network, dots, globules, and streaks.
Vascular pattern	Not present	Irregular hairpin vessels, dotted vessels, linear irregular vessels, or vessels within regression structures

**Table 2:** Melanoma clinical and histologic subtypes.

	%	Sun exposure	Localization	Clinical aspects	Colors	Histology
Superficial spreading melanoma	70	Intermittent	Back—Man Legs—Woman	Flat Papule Nodule	Tan, Brown, Gray, Black, Violaceous, Pink	Radial growth phase characterized by the proliferation within the epidermis of single atypical melanocytes in a pagetoid pattern

Nodular melanoma	5	Intermittent	Trunk Limb	Nodule Ulcerated polyp Elevated plaque	Brown Black Achromic	Radial growth phase absent

Lentigo maligna melanoma	4–15	Long term	Head Neck	Flat Papula	Brown Black	Radial growth phase composed of a lentiginous proliferation atypical melanocytes at the dermo-epidermal junction

Acral lentiginoso melanoma	5	N/A	Glabrous skin (palmoplantar, subungueal)	Flat Nodule Plaque	Irregular, poorly circumscribed pigmentation	Radial growth phase characterized by lentiginous proliferation of atypical melanocytes, marked acanthosis and elongation of the rete ridges of the epidermis

Desmoplastic melanoma	2	Long term	Head Neck	Papule Nodule	Erythemato us Flash colored Achromic	Vertical growth phase composed of atypical spindle cells embedded in a fibrous stroma

Melanoma arising from blu nevus	Rare	N/A	Head	Recent history of enlargement or change in pre-existing nevus	Blu-Black	Malignant dermal, deep seated component juxtaposed to a benign blue nevus

Melanoma arising in a giant	Rare	N/A	Trunk	Nodule growing in	Dark, brown	Dermal sharply delineated nodule

Congenital nevus				A nevus	Black	Composed of atypical ephitelioid, spindle or small cells, arising in a preexisting giant congenital nevus

Melanoma of childhood	0,4	N/A	Trunk	SSM or NM	SSM: Tan, Brown, Gray, Black, Violaceous, Pink	Conventional epithelioid melanoma or small cells or melanoma simulating Spitz nevus

Nevoid melanoma	1-2	N/A	Leg, trunk	Small papule	Tan to dark, brown	Nevus-like silhouette, limited intraepidermal component, loss/lack of maturation, cytological atypia, mitoses in the dermal component

**Table 3:** Instrumental investigation for clinical staging melanoma.

Pathologic features	Suggested tests
Melanoma in situ	None
Melanoma T1	Liver ultrasound Bilateral ultrasound of the lymph node drainage basin
Melanoma T2-T4a	Chest X-ray Liver ultrasound Bilateral ultrasound of the lymph node drainage basin
Melanoma T4b	Contrast-enhanced CT scan of the chest and abdomen Bilateral ultrasound of the lymph node drainage basin.

T1 ≤ 1 mm; T2 = 1,01–2,00 mm; T3 = 2,01–4,00 mm; T4 > 4,00 mm; a = not ulcerated; b = ulcerated.

**Table:** (a)

	Stage I		Stage II	
T				
	Not ulcerated	Ulcerated	Not ulcerated	ulcerated
0				
1	A	B		
2	A			B
3			A	B
4			A	C

**Table:** (b)

Stage III	
T	
Not ulcerated	Ulcerated
Metastasis 1 lymph node	Metastasis 1 lymph node
Micro A	Micro B
Macro B	Macro —

Metastasis 2 or 3 lymph node or intralymphatic	Metastasis 2 or 3 lymph node or intralymphatic
Micro A	Micro B
Macro B	Macro —
Intransit C	Intransit C

Metastasis 4/+ lymph node, matted metastatic node, in transit MT with MT in regional LN	Metastasis 4/+ lymph node, matted metastatic node, in transit MT with MT in regional LN
Micro —	Micro C
Macro —	Macro C

**Table:** (c)

Stage IV		
LDH	Normal	Elevated
M1		
Skin, subcutaneous, node	A	C
Lung	B	C
Any other	C	C

T1 ≤ 1 mm; T2 = 1,01–2,00 mm; T3 = 2,01–4,00 mm; T4 > 4,00 mm.

A-B-C: indicator of substaging; LDH: lactate dehydrogenase; M: metastases.
